# Precision cotton disease detection via transformer models applied to leaf imagery

**DOI:** 10.3389/frai.2025.1743264

**Published:** 2026-02-09

**Authors:** Nikhil Inamdar, Manjunath Managuli, Ramesh Koti, Jagadish Jakati, Sharanappa P. H., Prasan Kulkarni

**Affiliations:** 1 Department of Electronics and Communication Engineering, KLS Gogte Institute of Technology Belagavi and Affiliated to Visvesvaraya Technological University Belagavi Karnataka, Belagavi, India; 2 School of Computer Science Engineering & Applications Engineering, D. Y. Patil International University Pune, Pune, Maharashtra, India; 3 Department of Electronics and Communication Engineering, Basaveshwara Engineering College, Bagalkot, Karnataka, India; 4 Department of Electronics and Communication Engineering, Anuvartik Mirji Bharatesh Institute of Technology, Belagavi, Karnataka, India

**Keywords:** CNN, cotton plant, disease classification, image classification, transformer models

## Abstract

There is great potential for improving agricultural research, ecological monitoring, and biodiversity conservation through computerized plant species cataloging utilizing leaf photos. This work introduces a deep learning-based framework that uses transformer-based architectures, such as the Vanilla Vision Transformer (ViT), Swin Transformer, DeiT (Data-Efficient Image Transformer), and T2T-ViT (Tokens-to-Tokens Vision Transformer), to automatically classify cotton leaf diseases. Images of cotton leaves from four different classes—curl virus, bacterial blight, fusarium wilt, and healthy leaves—make up the dataset. A stratified K-fold hold-out testing technique (K = 1 to 5) is used to maintain the class distribution across training and testing folds in order to guarantee robust model evaluation and address class imbalance. To improve generalization and guarantee compatibility with transformer models, standard image augmentation and normalizing approaches are used. All models begin training using vast collections of images, afterward honed specifically on cotton leaf data to sharpen their ability to tell differences apart. Results spread across multiple test rounds stay steady, one standout reaching nearly perfect accuracy—99.99 percent. This pattern highlights how transformer-driven systems thrive alongside stratified K-fold checks, crafting a dependable way to spot crop issues early, shifting farm oversight toward quicker, smarter responses.

## 1 Introduction

In countries across South Asia—such as India, Bangladesh, and parts of southern China—farming still anchors daily life and livelihoods (Patil and Burkpalli, 2021; Nadiruzzaman et al., 2021). Yet shifting weather patterns brought on by warmer climates are worsening outbreaks of plant illnesses, chipping away at harvest yields. Cotton, often called white gold or nature’s silk, stands central among cash crops traded globally. Valued near 40 billion USD today, its market may climb toward 60 billion within this decade’s end (Khairnar and Goje, 2020; Meyer et al., 2023). India’s textile industry, valued at over 200 billion dollars, stands among the leading forces in global garment exports. Cotton, often seen as the backbone of this sector, grows here more than anywhere else on Earth. The nation leads worldwide in cotton production, fueling much of its fabric output. Rich soil and generations-old expertise help sustain this massive trade. From spinning yarn to finished garments, the work flows through countless hands across villages and cities alike.

India grows cotton across more than 12.9 million hectares, helped along by sunny weather and soil that suits it well. Still, growing this crop faces hurdles—blights creep in, bugs invade, rain plays tricks, dry spells hit hard, and temperatures jump without warning. Even so, countless farming families rely on it, pulling through because it keeps food on the table and work in their hands. In China, sicknesses and insects chew away at harvests every year, shaving off 15 to 20 percent of value, sometimes even half when things go wrong. Most troubles plaguing Indian cotton show up first on leaves, dragging yields down by one out of four. Spotting those signs sooner could rescue roughly every fifth plant touched by illness, showing how much depends on catching trouble fast. In places such as Bangladesh and India, farmers often spot cotton problems by sight—sometimes aided by specialists. Without enough training, visual checks tend to fall short, demand heavy effort, yet remain common across remote regions. After spotting an issue, growers typically turn to chemical sprays, following guidance from advisors (Gupta and Pathak, 2016; Chen et al., 2020). Snapshots of leaves, pulling out distinguishing traits, then analyzing them form the core of automatic detection tools. Because they excel at uncovering meaningful patterns, methods rooted in computer vision—especially deep learning (DL) and machine learning (ML)—have drawn growing interest in studies (Talukder et al., 2023; Saleem et al., 2021; Dhaka et al., 2021).

A study introduced a way to spot sick soybean leaves by combining k-means grouping with SVM classification (Kaur et al., 2018). It reached 90% correctness across 4,775 images, targeting issues like bacterial pustule, blight, and mildew—using color plus surface patterns to tell healthy from infected. As deep learning advanced, various tests revealed transformers and convolutional networks do better at spotting and outlining crop diseases, especially if plenty of labeled examples exist (Talukder et al., 2022; Uddin et al., 2023). In particular, Vision Transformers can grasp focused traits through pre-trained knowledge, working well even when only small or niche image sets are accessible. That trait makes them fit for farming uses where data often runs short.

### 1.1 Key research findings are enlisted as below

#### 1.1.1 DL for cotton leaf disease detection

- The learning best part the efficiency of modernizer models like Vanilla Vision Transformer (ViT), Swin Transformer, DeiT, and T2T-ViT in classifying cotton leaf diseases.
- These models accurately differentiate four categories: curl virus, fusarium wilt, healthy leaves, and bacterial blight.

#### 1.1.2 High classification accuracy

- The model’s performance was enhanced by hyperparameter optimization, reaching an exceptional peak accuracy of 99.99%.

#### 1.1.3 Potential for early disease diagnosis

- The research shows these tools can spot cotton leaf issues sooner—important when quick farming decisions matter. By catching problems earlier, farmers gain time to act before damage spreads through crops.

#### 1.1.4 Contribution to agriculture and ecology

- The approach pushes progress in farm automation while boosting nature protection—woven together through smart tech that learns on its own.

## 2 Literature survey

Photos snapped under perfect farm conditions help spot plant illnesses—researchers have explored this angle plenty. Take wheat rot detection: it demanded shots from above plus close-ups across fields (Safari et al., 2022). Before being entered into an organization system to identify the precise class, these photos were annotated for object recognition, with bounding boxes defined and cropped. Five arrangement models utilizing CNN architectures were developed, including VGG16 (Simonyan and Zisserman, 2014), ResNet-50 (He et al., 2016), Inception (Szegedy et al., 2016), MobileNet-V3 (Howard et al., 2019), and EfficientNet-B0 (Tan and Le, 2019). Out of the five, EfficientNet-B0 proved to be the most accurate and computationally efficient prototype. Convolutional Neural Networks (CNNs) have become more popular in the identification of plant diseases as AI technologies have advanced. For example, in a comparison analysis utilizing the Plant Village dataset, the DenseNet model, which is well-known for its capacity to recycle article maps, achieved a noteworthy precision of 98.27% (Akshai and Anitha, 2021; Hughes and Salathe, 2015).

An average F1-score of 95.70% has been attained for apple leaf disease recognition training using ResNet networks with residual structures (Yu et al., 2022). By eliminating crucial elements from photos, CNNs are quite successful in mechanically categorizing plant illnesses. CNNs’ parameter-sharing method, which reduces the number of constraints and overfitting—a prevalent problem in computer vision tasks—is a major advantage. However, there is a chance of unnecessary computational cost as the system’s depth increases. Furthermore, CNNs do not explicitly use pixel positioning information, which may limit their capacity to grasp spatial relationships in the image, even when they are successful at extracting local area features through convolutional layers.

Conventional CNNs may be less successful in identifying plant diseases because they frequently have trouble utilizing pixel positional information. Vision Transformers (ViTs), first presented in Dosovitskiy et al. (2020), use a self-attention mechanism as suggested in Vaswani et al. (2017) to get around this. Diverse feature extraction from images is made possible by improvements such as incorporating a ghost module into the ViT encoder, as investigated in Lu et al. (2022). Additionally, the MSCVT model presented in Zhu et al. (2023) greatly advances the identification of agricultural diseases by combining the advantages of multiscale convolution and self-attention in a hybrid CNN-ViT architecture.

These research ignored tea leaf datasets even though they showed great accuracy using publicly available datasets. ICVT (Inception Convolutional Vision Transformer), which combines the Beginning construction with cross-channel article information, was developed by Yu et al. (2023) to close this gap. Additionally, Thakur et al. (2021), Alharbi et al. (2023), Dhakal et al. (2023), Jenifa et al. (2019), Nigam et al. (2023), Prakash and Managuli (2024a), Managuli et al. (2024), Prakash and Managuli (2024b), and Nikhil et al. (2024) introduced PlantViT, a transformer-based method specifically designed for accurate plant disease detection.

Researchers explored classic machine learning along with deep learning approaches to spot plant illnesses across multiple projects. Take study (Kaur et al., 2018), which introduced an approach for detecting issues on soy leaves through color patterns and texture traits. This method combined k-means grouping with SVM models to classify problems such as downy mildew, bacterial blight, plus frog-eye. It reached 90 percent precision when tested against a collection of 4,775 images.

The strong pattern recognition skills of CNN-driven designs have pushed their broad use in sorting plant illnesses through deep learning frameworks (Talukder et al., 2022; Uddin et al., 2023). Yet even with success, these networks can stumble under uneven light, messy surroundings, or when sickness signs appear across scattered parts of leaves. Lately, researchers have begun exploring transformer structures for visual challenges. Thanks to wide-ranging focus mechanisms and an ability to build rich contextual insights, models like ViTs, Swin Transformers, DeiT, and T2T deliver promising outcomes across various image classification areas. Still, applying them to detect crop diseases remains limited—especially on data covering multiple species.

### 2.1 Research gap

Despite progress in automated plant disease detection, gaps remain. While attention has shifted toward newer methods, studies focusing on transformer models for crop illness classification stay sparse—particularly those leveraging diverse, multi-species data. Most prior work leans on CNNs or traditional algorithms, which may overlook extended visual patterns crucial when symptoms look nearly identical across types. Rarely do papers line up ViT, Swin, DeiT, and T2T side by side under one testing framework. Cross-species consistency within shared disease families gets little attention. Evaluation often skips thorough metric analysis, tuning depth, or uniform preprocessing steps.

## 3 Methodology

**Figure 1:**
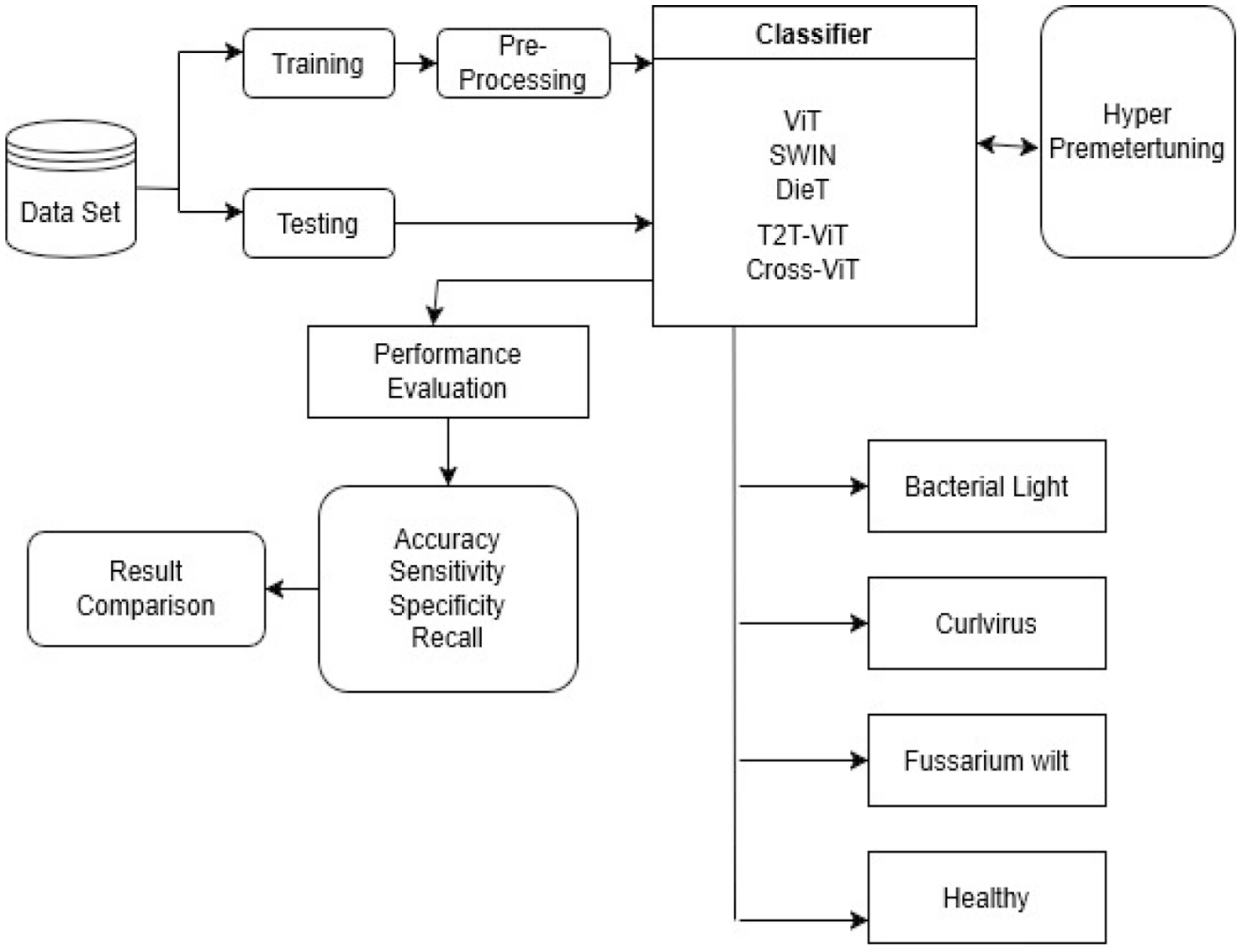
Block diagram of the proposed study.

Figure 1 displays the framework layout of the proposed method. The entire structure consists of four consecutive steps, each built to enhance precision and robustness in the organizational process. First comes pre-processing—then deep feature extraction follows. After that, features undergo refinement through optimization. Finally, classification takes place as the last phase.

### 3.1 Pre-processing

To enhance data excellence and guarantee consistency throughout the dataset, the input photos go through pre-processing in the first step. This stage handles tasks like boosting contrast, resizing images, smoothing values, while cutting down random interference. Cleaning things up here removes distractions that could confuse later steps where patterns start to form.

### 3.2 Extraction of deep features

Once cleaned up, the visuals move into a deep learning setup where features start to emerge. Through a trained or tailored neural net—say, a CNN or maybe a Vision Transformer—the system picks out distinct, high-layer traits on its own. Instead of handcrafting details, it zeroes in on subtle shapes, surface variations, and layout clues that matter for telling one class apart from another.

### 3.3 Optimization of features

The refinement stage sharpens the deeply pulled features further. Removing clutter happens through techniques like PCA, smart search strategies, or rank-based filters—cutting what is not needed. With fewer distractions, the remaining traits stand out better. Overfitting slips away as complexity drops. Less bulk means faster processing, lighter load.

### 3.4 Classification

In the final stage, a model takes the refined features to reach distinct decisions. Instead of simple grouping, intelligent patterns guide how inputs are sorted through methods like SVMs, neural nets, or softmax logic. Outcomes emerge directly from these sorting results, shaping what the system ultimately predicts.

**Figure 2:**
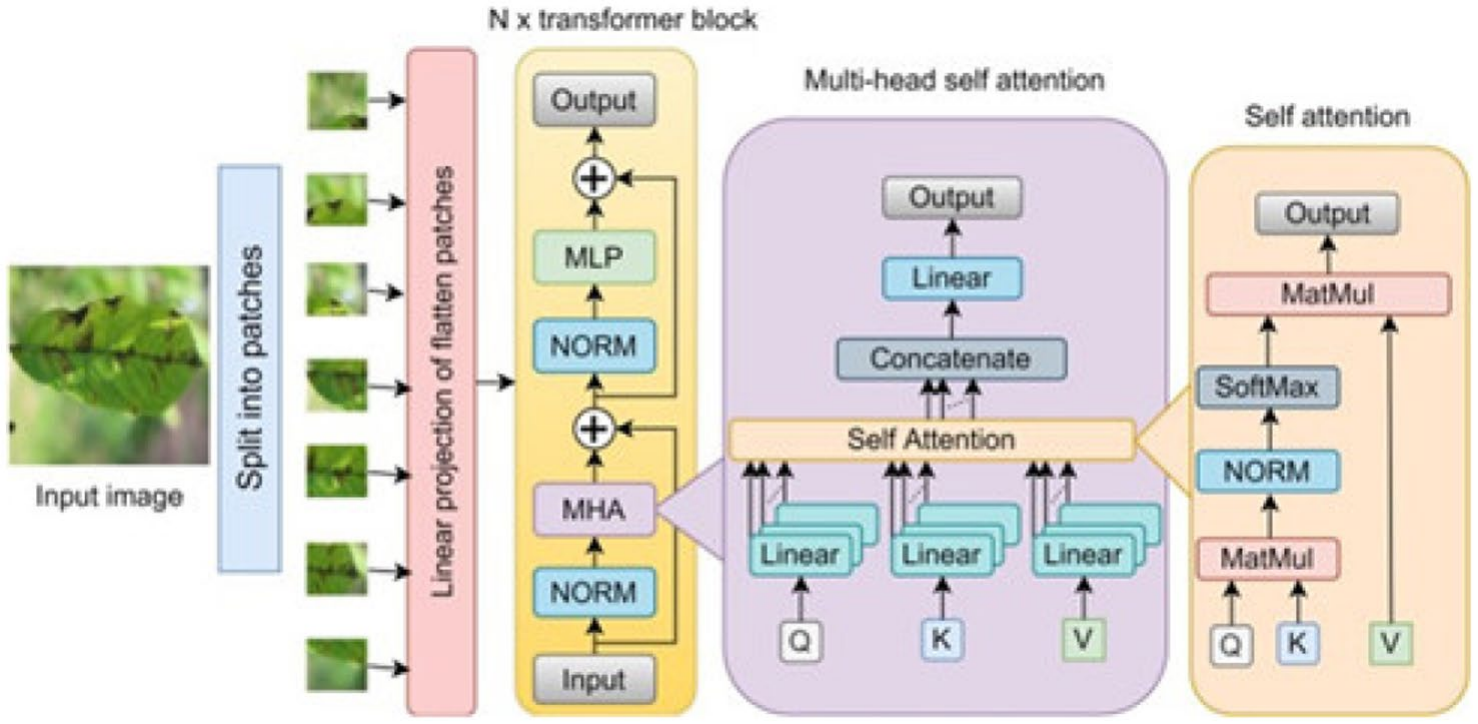
Block schematic of vanilla vision transformer model.

**Figure 3:**
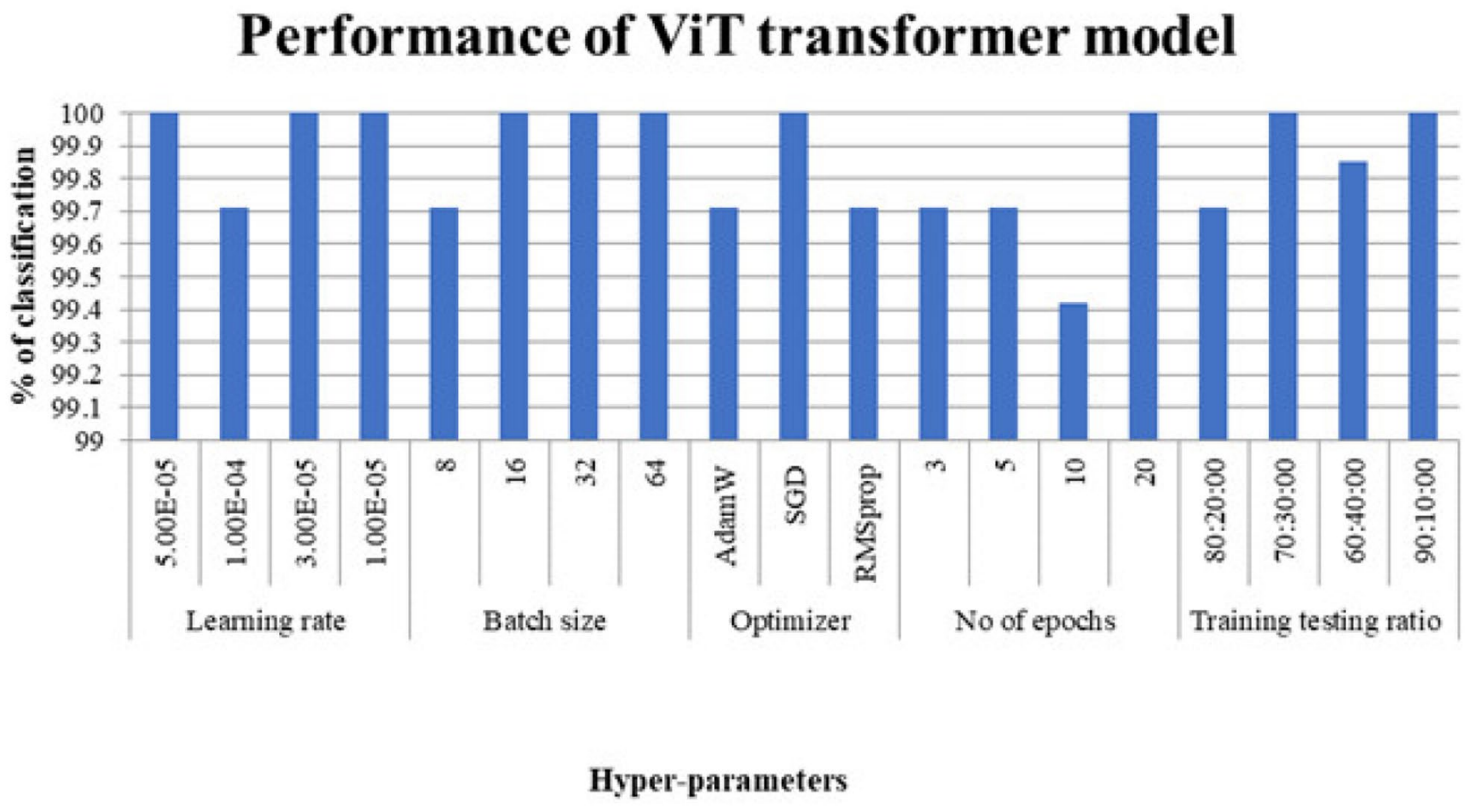
Classification accuracy considering ViT transformer models.

The manuscript points to the performance as Figure 2—though it’s really labeled Figure 3. Since mismatches happen, every figure reference should still be double-checked.

### 3.5 Dataset

This research targets the organization of cotton plant leaf diseases using transformer models, an area with limited prior exploration compared to other crops. A diverse dataset of leaf images, sourced from studies cited in the literature survey, is utilized. Sample images and associated counts are given in Table 1, highlighting the dataset’s composition for effective model training and evaluation.

**Table 1:** Sample images from the dataset used.

Sl no	Disease type	Image	No. of images
1	Bacterial blight	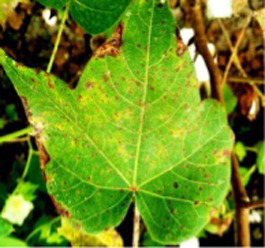	448
2	Curl virus	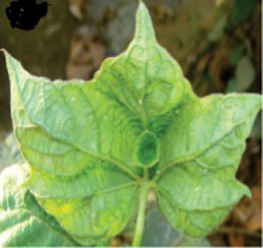	418
3	Fussarium_wilt	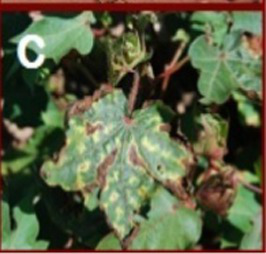	419
4	Healthy	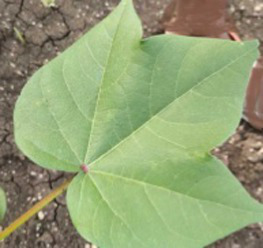	426

Datasets. Rather than collecting new material, this work pulled data from a shared online archive—boosting transparency while supporting consistent method evaluation across trials. Its 1,711 images fall into four distinct groups, though some count them as five. Every category includes several samples used for testing and teaching the system. Since every picture came from identical setups, their sharpness and visual clarity matched throughout.

### 3.6 Pre-processing

The dataset featured leaves from four distinct plants, each image first checked by hand to toss out blurry or broken ones. After sorting, every photo got scaled down—no exceptions—to fit neatly into 224 by 224 pixels. This standard frame helped the system process visuals without hiccups. Pixel values were then shifted, gently compressed into a range between zero and one, smoothing the path for steady learning. Keep in mind, the dataset pulls together images from four distinct plant groups—yet these serve only as origins. Instead of sorting by plant kind, the task focuses on grouping leaves by sickness type. Each category blends specimens from multiple species, tied not by genus but by symptom patterns. This setup pushes the model to pick up on signs of illness that appear across different plants. By mixing varieties within each disease group, it learns traits that stretch beyond a single host.

### 3.7 Classifiers

A handful of familiar vision transformers—Swin, DeiT, T2T-ViT—took part in this research; yet the real twist lies in their head-to-head testing across diverse plant species and diseases. Instead of scattered methods, one consistent pipeline handled both prep and analysis, stitching fairness into comparison. Rare in farm-focused machine learning circles, such an approach builds a reproducible frame other can step into. What emerges is not just rankings, but clearer views on which architectural flavors suit sick leaves best.

### 3.8 Vanilla vision transformer model

**Figure 4:**
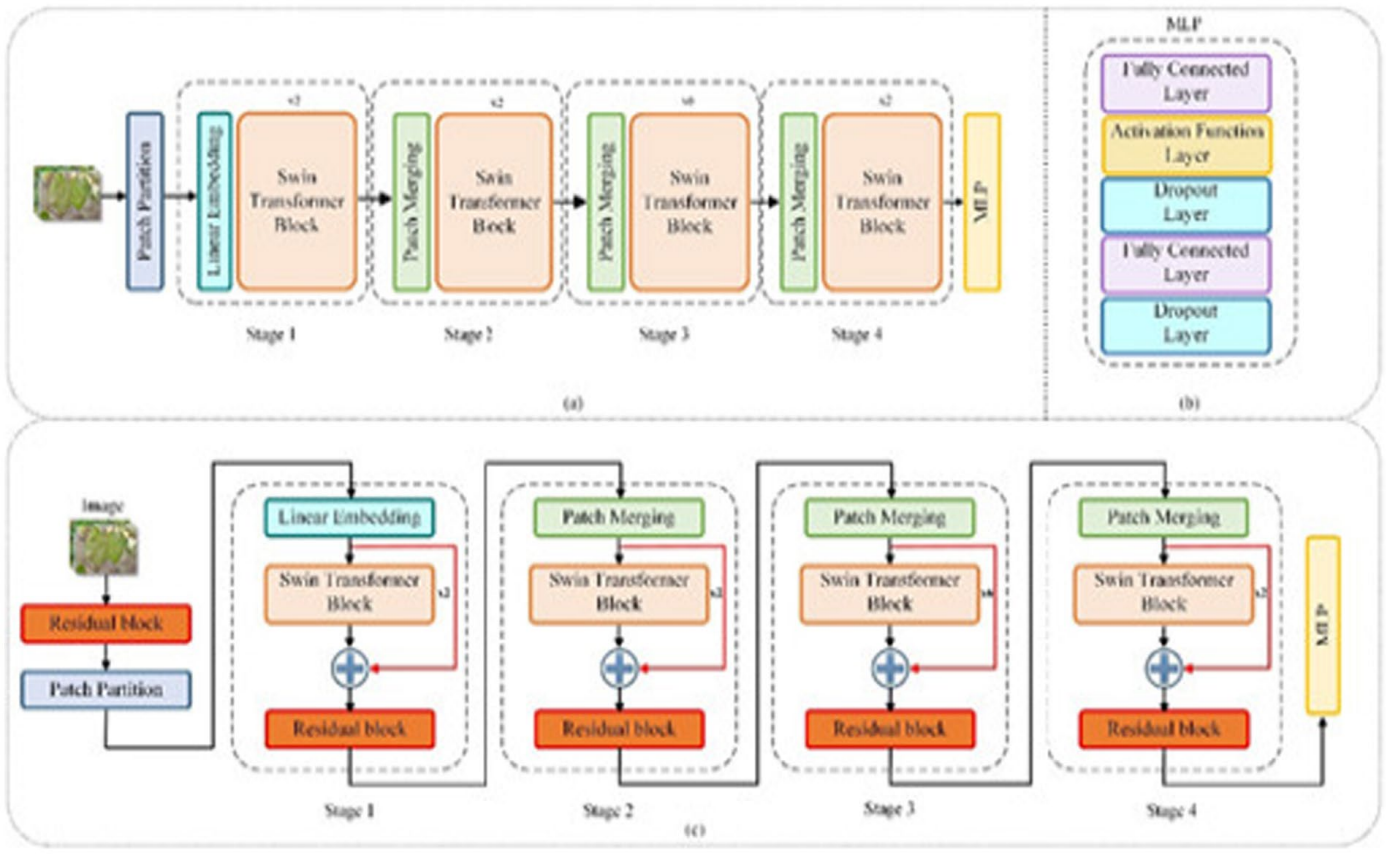
Block schematic of swin transformer model.

Figure 4 shows the architecture of a Vision Transformer (ViT), a deep learning model designed to process images by applying transformer architecture. Let us break down each part of the Figure 4 diagram:

1. Input Image and Patch Splitting: The input appearance is separated into slighter, fixed-size covers, each compressed into a vector & treated as a separate “token,” similar to words in NLP models.
2. Linear Projection of Flattened Patches: Apiece flattened patch is expected into an implanting interplanetary, starting an order of embedding, which serves as input to the transformer blocks (similar to word embedding in NLP).
3. Transformer Blocks (repeated N times): The ViT architecture consists of numerous modifier blocks, each with the following components:

- Multi-Head Self Attention (MHA): Multiple attention heads calculate attention scores, capturing interactions among patches.
- Normalization (NORM): Normalization layers are applied before and after attention layers to stabilize training.
- MLP (Multi-Layer Perceptron): Binary bits with a starting meaning are processed by a feedforward neural network.
- Residual Connections (+): Additional skip connections between layers improve gradient flow and stop the vanishing gradient issue.

#### 3.8.1 Multi-head self attention (MHA) block

The MHA block includes multiple self-attention “heads,” which help the model capture different aspects of relationships between patches.

- Q, K, V (Query, Key, Value): These are linear transformations of the input patch embeddings, which are used to calculate attention scores between different patches.
- Concatenate and Linear: The output from each attention head is concatenated and passed through a linear layer to combine information from all heads.

#### 3.8.2 Self-attention block

This block shows how self-attention is calculated within each head.

- MatMul and SoftMax: The Query and Key vectors are multiplied (MatMul) to calculate attention scores, and SoftMax is applied to normalize these scores.
- Output Calculation: The attention scores are then used to weigh the Value vectors, generating the final output for each attention head.

Figure 2 shows the performance of the Viet modifier for the dataset considered.

#### 3.8.3 Swin transformer

**Figure 5:**
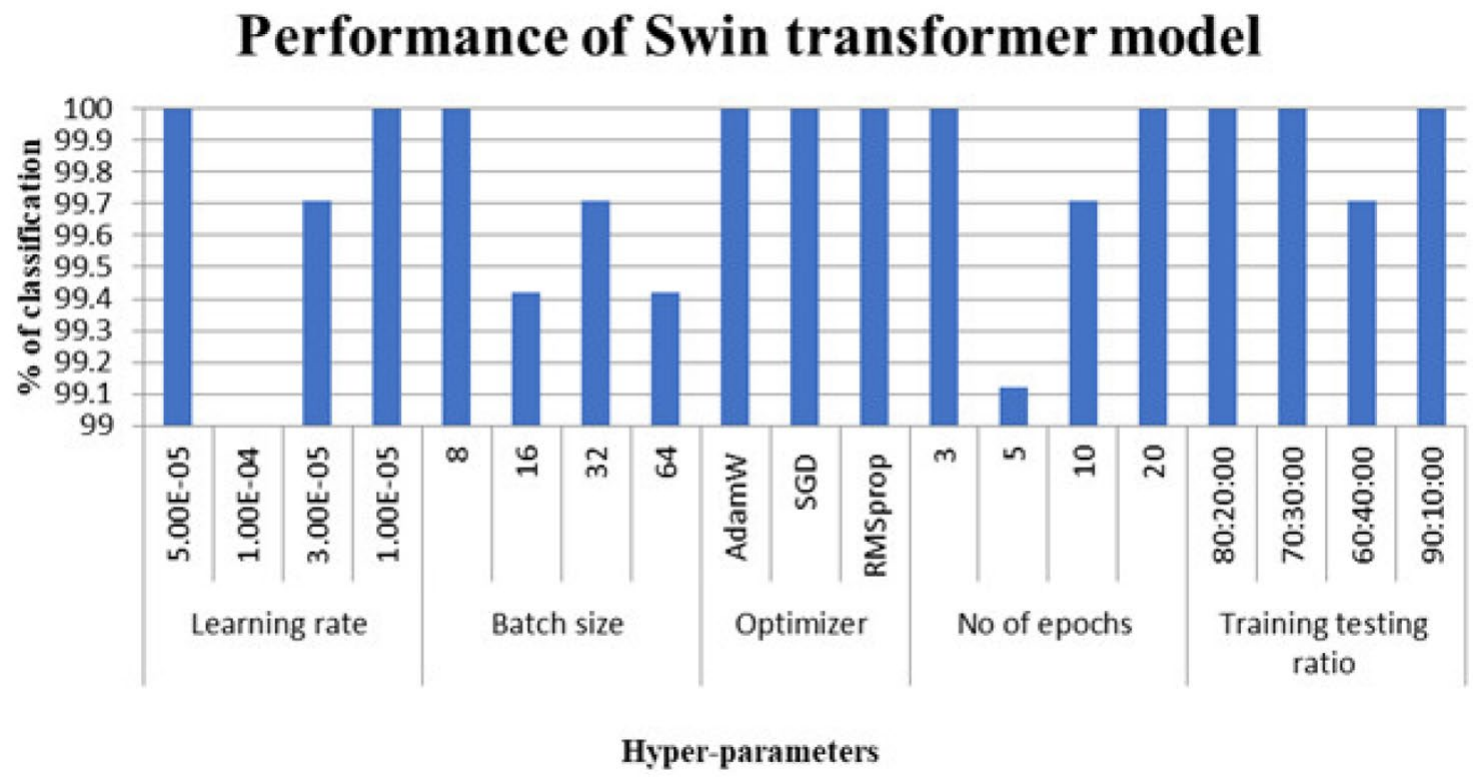
Classification accuracy considering Swin transformer models.

This image illustrates the architecture of a Swin Transformer, a type of Vision Transformer specifically designed for handling visual tasks in a hierarchical and efficient manner. The architecture diagram, as shown in Figure 5, is divided into two main parts, showing two configurations of the Swin Transformer. Let us go through each section:

#### 3.8.4 Overview of Swin transformer stages

1. Patch Partitioning: The input appearance is separated into covers, where each patch represents a small region of the image.
2. Linear Embedding: Each patch is compressed and linearly predictable to make a lower-dimensional embedding, similar to the process in Vision Transformers.
3. Stage Processing with Swin Transformer Blocks: The architecture is organized into four stages, each with a different number of Swin Transformer blocks:
  - Swin Transformer Block: Each block processes the input embedding using window-based self-attention, where attention is computed within local windows instead of the entire image.
  - Patch Merging: After each stage, patch merging reduces the number of patches by combining neighboring patches, increasing the receptive field as the network deepens. The number of Swin Transformer blocks increases in each stage, allowing for more complex representations.
4. MLP Layer: After the fourth stage, the mined topographies are approved through an MLP skull, consisting of completely linked layers, beginning functions, and dropout for classification tasks.

Figure 3 shows the performance of the swin converter for the dataset considered.

#### 3.8.5 DeiT (data-efficient image transformer)

The DeiT is a variation of the ViT designed to recover the replica’s competence and accuracy, especially when training on smaller datasets Created in 2021 by Facebook AI, DeiT rolled out methods that ease reliance on huge data loads typical of standard transformers—suddenly, large-scale pre-training wasn’t a strict requirement. Instead of leaning on vast datasets, it leaned into smarter training tricks. These opened doors for wider adoption, especially where computing power or data access is limited. Efficiency became its quiet strength.

#### 3.8.6 Distillation token

- The DeiT setup adds a distillation token along with the usual CLS marker—two tokens moving through layers together, each playing distinct roles during training. One guides label-based learning, while the other absorbs knowledge from a teacher network, shaping how features evolve across blocks.
- A teacher model, often a CNN or large pretrained transformer, guides the student during training through the distillation token, shaping its progress more effectively while streamlining knowledge transfer.
- Through smooth knowledge sharing, the teacher model offers fuzzy guesses for each category—helping DeiT adapt better when data is limited.

1. Training on Smaller Datasets:

- DeiT works better when data is limited, whereas the first ViT relies on massive sets—JFT-300 M or ImageNet-21 k—to learn effectively beforehand
- A single million-image dataset like ImageNet-1 k can still push DeiT forward—especially once the distillation token steps in, trimming reliance on massive data loads while holding its own in results.

2. Multi-Head Self-Attention and Feedforward Layers:

- Similar to ViT, DeiT maintains the basic transformer structure with multi-head self-attention and feedforward layers.
- Its typical architecture uses positional encodings, fixed-size picture patches, and a number of transformer encoder layers.

3. Efficient Training Techniques:

- To lessen overfitting, DeiT uses a number of training improvements, including data augmentation, regularization, and stochastic depth, which randomly removes pathways from the model during training.

4. Loss Function:

- DeiT combines the standard cross-entropy loss with a distillation loss. The cross-entropy loss is computed for the organization token, while the distillation loss is computed for the distillation token, based on the teacher’s predictions.
- This dual-loss approach encourages the model to balance both the actual label predictions and the soft label guidance from the teacher.

**Figure 6:**
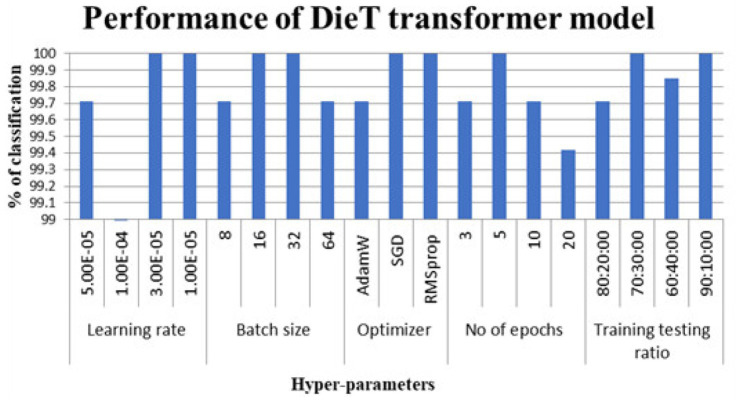
Classification accuracy considering DieT transformer models.

Figure 6 shows the performance of the swin modifier for the dataset considered.

#### 3.8.7 Token to token transformer

**Figure 7:**
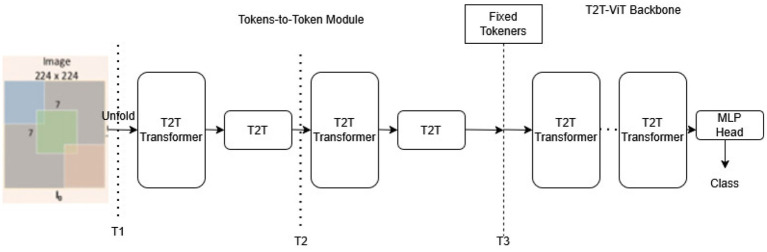
Block schematic of T2T transformer model.

Figure 7 depicts the architecture of the T2T-ViT (Tokens-to-Tokens Vision Transformer), which is designed to enhance the Vision Transformer (ViT) by capturing better local structure information within the input images.

Here’s a breakdown of the architecture as shown in the diagram:

1. Input Image (224 × 224 pixels):

- The T2T-ViT model begins with an input image of size 224 × 224 pixels.
- To capture additional spatial information in each location, this image is then segmented into overlapping patches rather than normal non-overlapping patches.

2. Tokens-to-Tokens (T2T) Module:

- The input image is converted by the T2T module into tokens, which are the basic units supplied to the transformer.
- It operates in multiple stages to progressively merge tokens, thereby creating a hierarchy of tokens that better represents the image’s local information.

3. Transformer Backbone:

- After processing in the T2T module, the tokens are passed to the main Transformer backbone.
- This backbone consists of various transformer layers, each with multi-head self-attention mechanisms and feed-forward networks.
- Each layer processes the tokens, allowing the typical to imprisonment worldwide situation and relations across different image regions.
- Positional Encoding (PE): To maintain spatial information, positional encodings are added to the tokens since transformers alone do not inherently understand spatial positioning.

4. MLP Head:

- After passing through the transformer layers, the tokens are directed to the MLP Head for organization.
- The output from the MLP head classifies the image into one of the target categories in a classification task.

**Figure 8:**
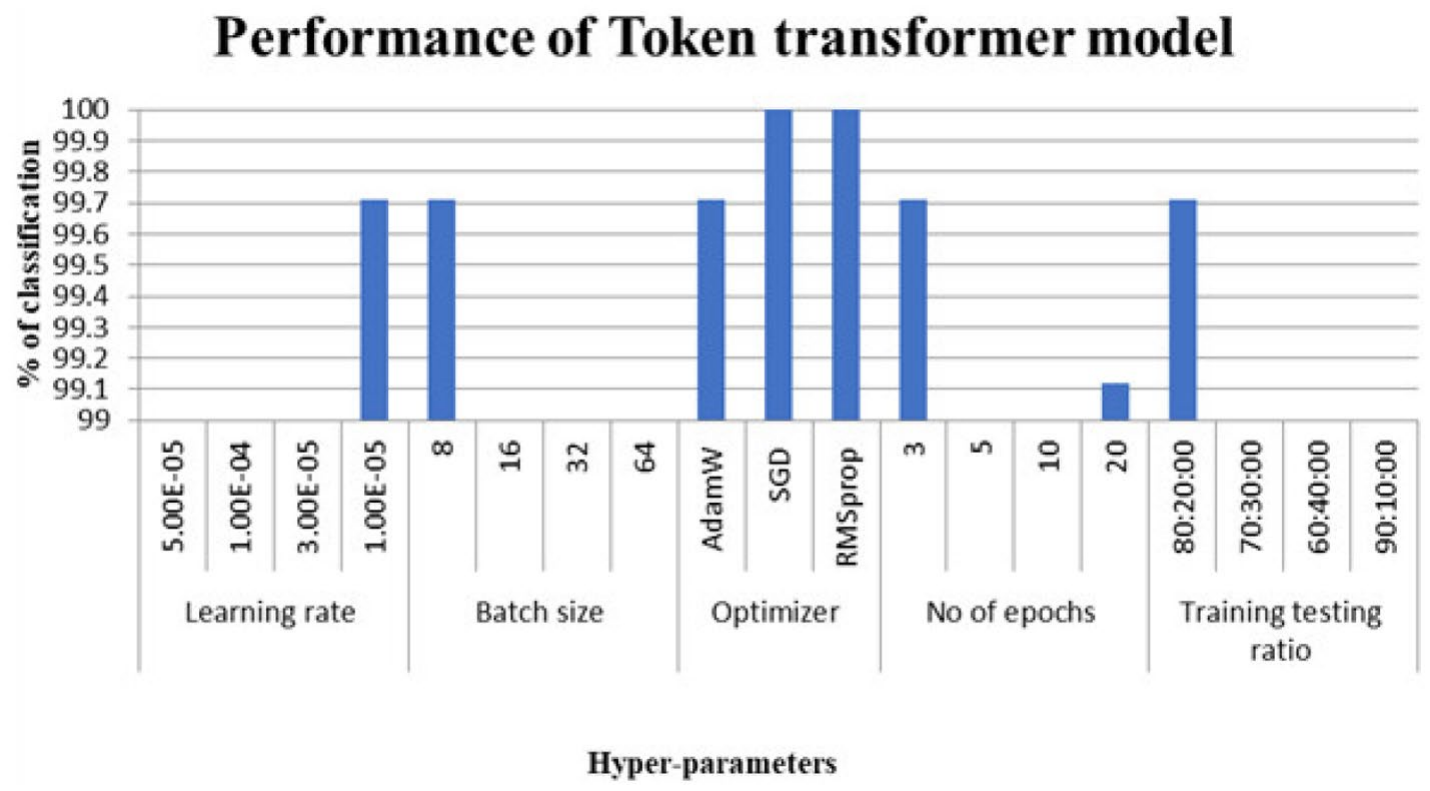
Classification accuracy considering T2T transformer models.

**Figure 9:**
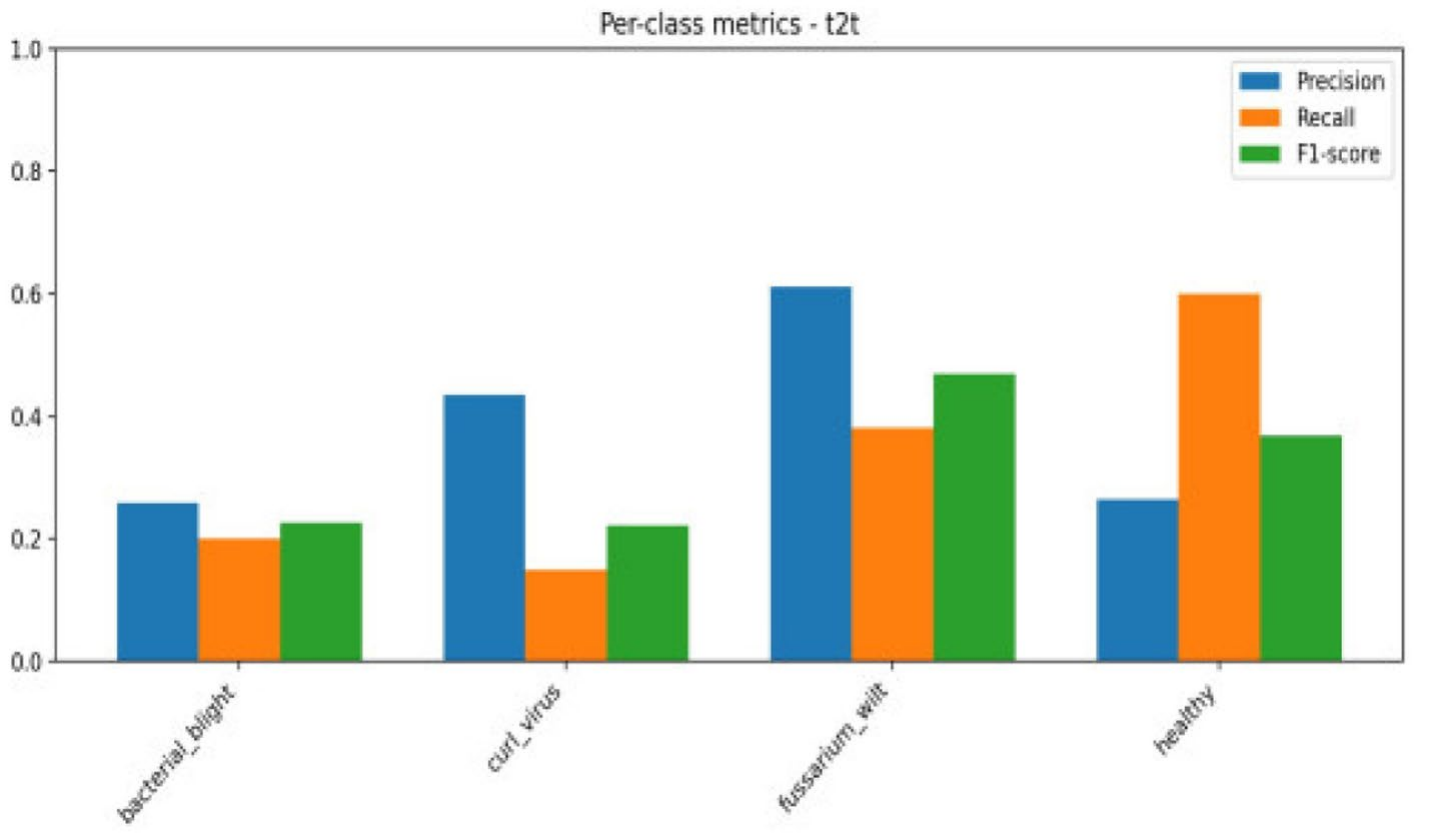
T2T matrices.

Figures 8, 9 show the performance of the T2T transformer for the dataset considered.

## 4 Results and discussion

Instead of depending only on a single train-test split, the typical approach was further validated to guarantee a more trustworthy and objective performance evaluation. Because the model is tested across several data partitions, this method offers a more thorough assessment of model stability and generalization. The average performance over all folds is represented by the final reported results.

Such automated disease detection systems can possibly enable large-scale crop health monitoring and contribute to broader agricultural sustainability efforts, even if the main focus of this education is the categorization of cotton leaf diseases using transformer-based models. However, ecological monitoring and biodiversity conservation are only listed as potential uses for further research; they are not directly evaluated.

Preprocessing steps, including image augmentation and normalization, ensure consistency and quality for model input. Transformer models such Vanilla Vision Transformer (ViT), Swin Transformer, DeiT, and T2T-ViT are used; hyper-parameter tuning is used to determine which model performs the best. To demonstrate the superiority of the suggested method, performance is assessed using common classification criteria, and findings are contrasted with those of current research. To examine model behavior and effectiveness in cotton leaf disease detection, extensive trials are carried out, backed by confusion matrices and comprehensive classification reports.

The hyperparameters that led to perfect classification on this particular dataset appear in Table 2. Despite their earlier mention in the methodology, results for the token-to-token transformer were left out of Table 3—accuracy fell short of the full mark.

**Table 2:** Classification report.

Name	Precision	Recall	F1-Score	Support
Curl Virus	0.84	0.81	0.83	315
Bacterial Blight	0.85	0.83	0.84	335
Fusarium wilt	0.92	0.93	0.93	325
Healthy	0.82	0.86	0.84	320
Accuracy			0.86	1,295
Macro avg	0.86	0.86	0.86	1,295
Weigted avg	0.86	0.86	0.86	1,295

**Table 3:** Hyper parameters selected for optimal performance.

Name	ViT	Swin	DeiT
Learning rate	1.00E-05	1.00E-05	1.00E-05
Batch size	16	8	16
Optimizer	SGD	SGD	SGD
No of epochs	20	20	5
Training testing ratio	70:30	71:30	72:30

**Figure 10:**
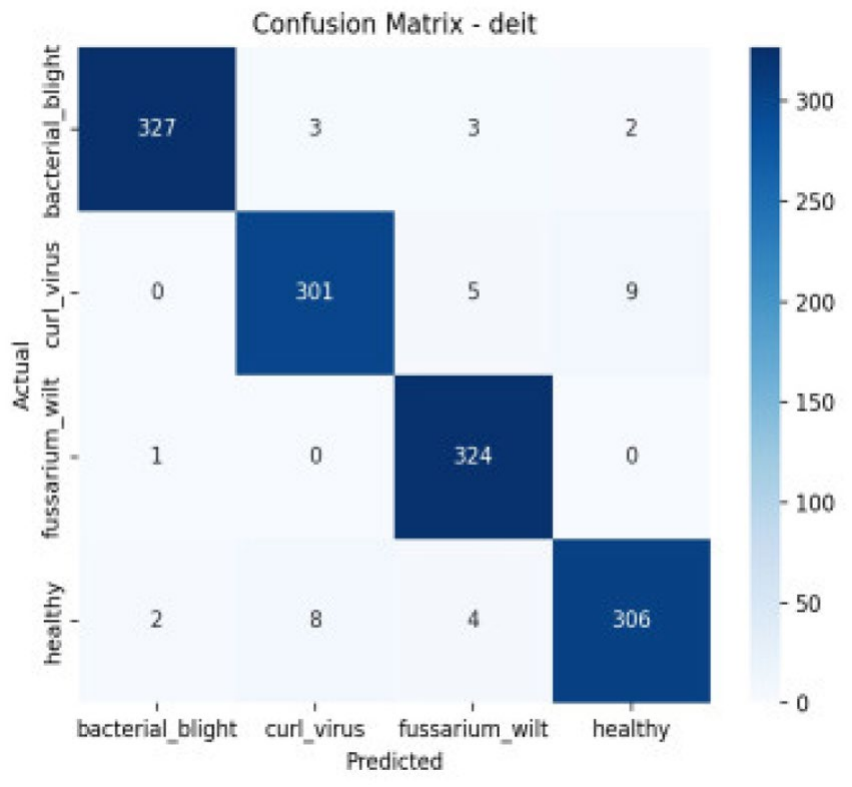
The classification report’s confusion matrix.

**Figure 11:**
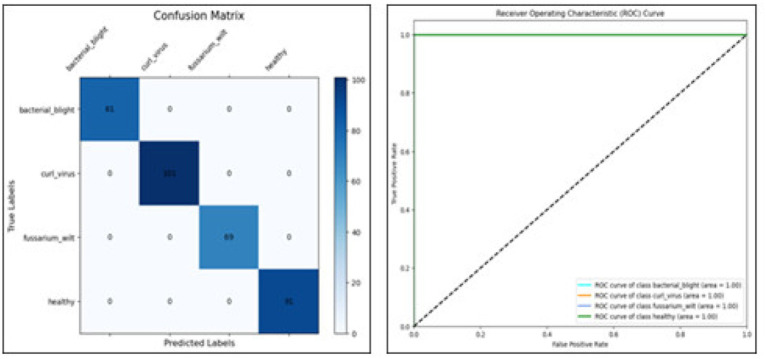
Confusion matrix and RUC curve of transformer models.

The confusion matrix breaks down the prototype’s performance, sorting outcomes into true negatives, true positives, along with misjudged cases—false negatives and false positives. These categories spotlight where errors tend to cluster. Meanwhile, the ROC curve shows how sensitivity shifts against false alarms as thresholds change. Greater AUC points toward clearer separation between classes. The AUC reflects how well a model performs across thresholds. As shown in Table 4, Figure 10 together with Figure 11 displays confusion matrices alongside ROC plots for multiple transformer setups paired with specific classifiers—revealing uneven results in classification tasks.

**Table 4:** Classification report of Deit.

Name	Precision	Recall	F1-Score	Support
Curl Virus	0.99	0.98	0.98	335
Bacterial Blight	0.96	0.96	0.96	315
Fusarium wilt	0.96	1.0	0.98	325
Healthy	0.97	0.96	0.96	320
Accuracy			0.97	1,295
Macro avg	0.97	0.97	0.97	1,295
Weigted avg	0.97	0.97	0.97	1,295

**Figure 12:**
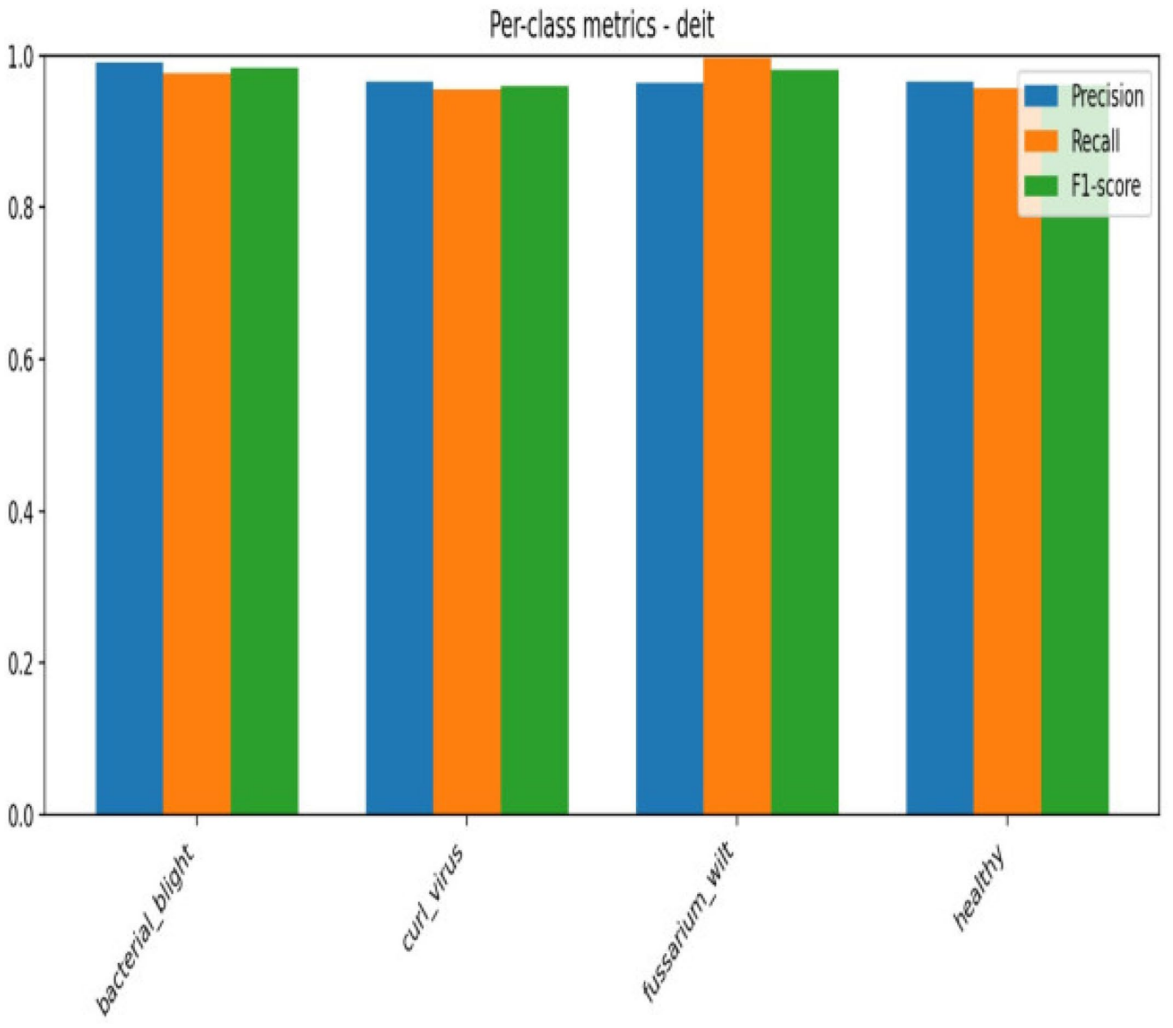
Per class metrics-Deit.

**Figure 13:**
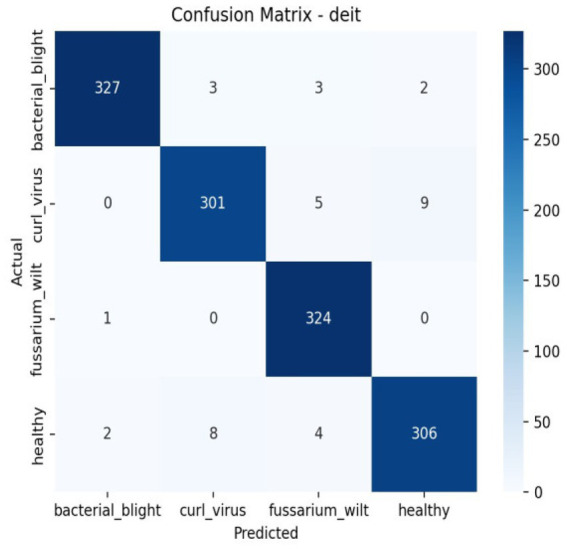
Confusion matrix Deit.

Although accuracy offers a general indicator of accurate predictions, it might not adequately represent how the model behaves in other classes. Thus, we assessed the model using precision, recall, F1-score, confusion matrix, and ROC-AUC in addition to accuracy. The F1-score balances both metrics, the confusion matrix offers comprehensive insights into misclassification patterns, and precision and recall aid in evaluating class-wise performance. These extra criteria guarantee a more thorough and dependable evaluation of the model’s performance in Figures 12, 13.

**Figure 14:**
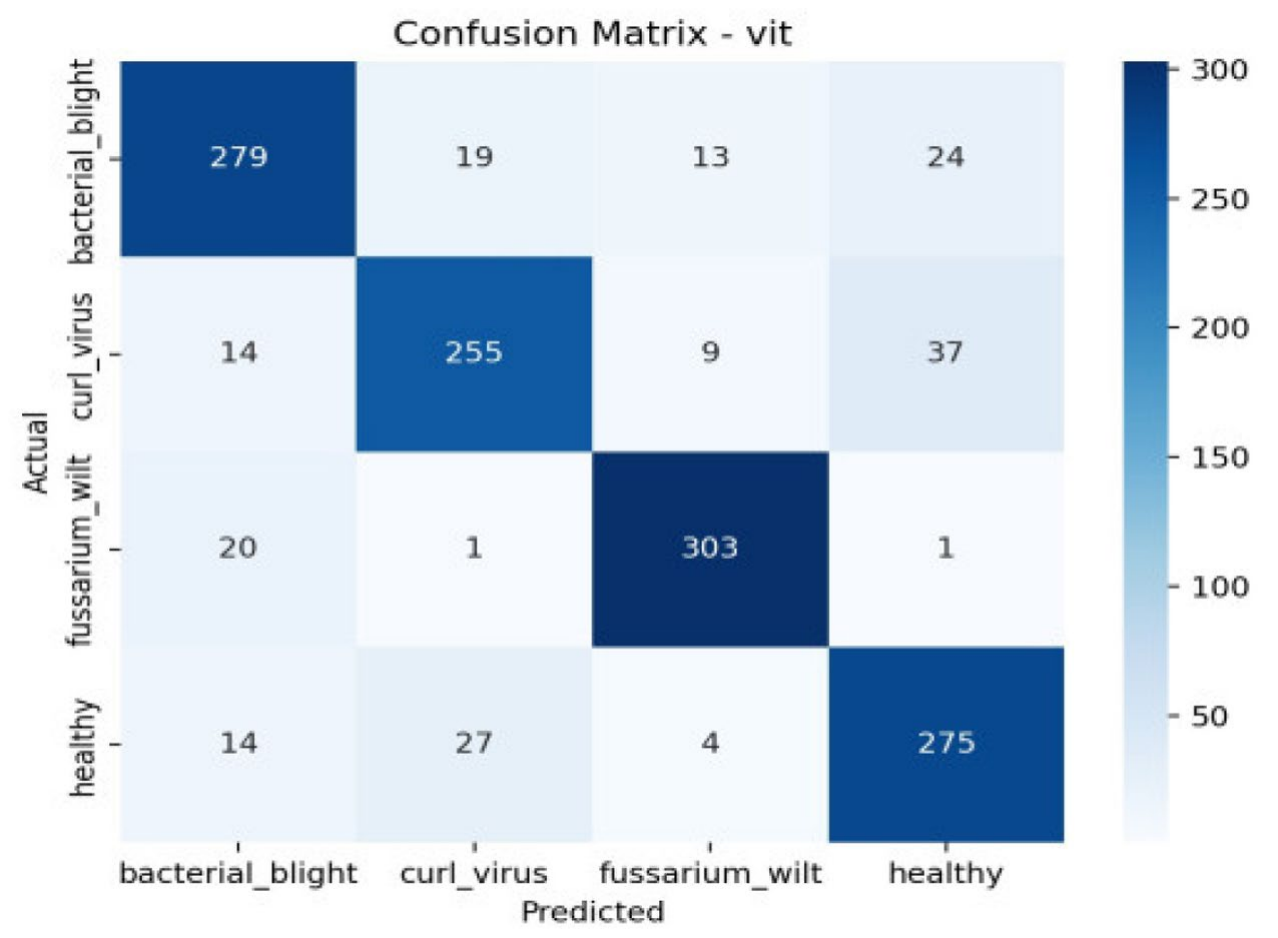
Confusion matrix of ViT.

There are a number of reasons why the transformer-based versions perform better. First, self-attention methods used by transformer architectures like ViT, Swin Transformer, DeiT, and T2T enable the model to extract global contextual information and long-range dependencies from leaf images. Because disease indicators, including discoloration, texture changes, and uneven patterns, are frequently dispersed among several leaf sections, this ability is especially helpful for disease diagnosis Figure 14.

Second, transformers dynamically learn associations between all picture patches, allowing for better feature representation and enhanced disease class discrimination, in contrast to typical CNNs that rely on fixed-size convolutional kernels. Higher resilience results from this, particularly in datasets where different plant species may exhibit comparable illnesses.

**Figure 15:**
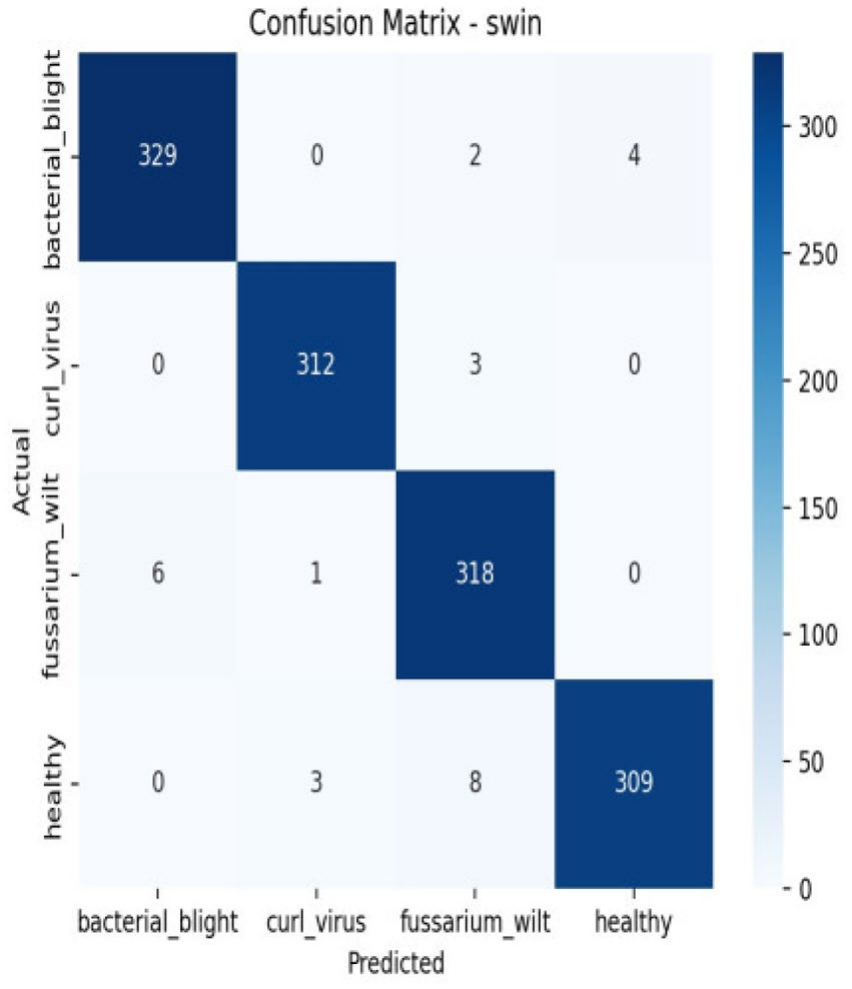
Confusion matrix of Swin.

**Figure 16:**
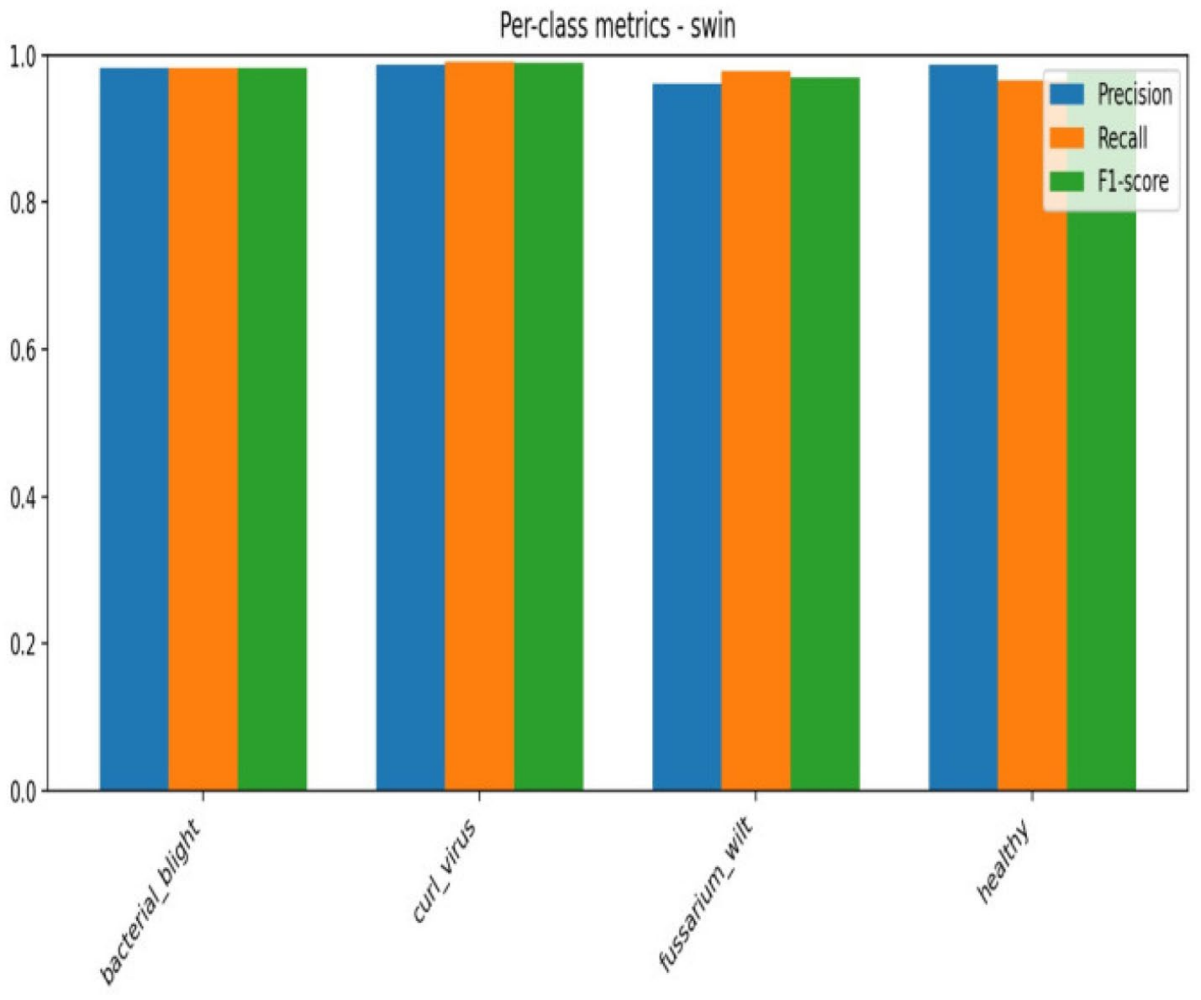
Per class metrics-Swin.

Third, the remarkably high accuracy attained in our tests is a result of the enhanced pre-processing procedures and hyper parameter tuning, which further improve model generalization. Additionally, patch embedding and multi-head attention give transformer models strong regularization capabilities that assist in minimize overfitting even when working with a complex dataset that includes 4 different leaf types in Figures 15, 16.

### 4.1 Discussion of the proposed model

The dataset utilized in this study comprises 1,711 images categorized into four classes of cotton plant leaf diseases. Convolutional neural networks (CNNs) and other traditional machine learning and deep learning techniques have been widely investigated for automated plant disease identification using leaf imagery. Although CNN-based models have shown encouraging results, their dependence on limited receptive fields frequently restricts their capacity to grasp global contextual information and long-range dependencies found in intricate leaf disease patterns. Hybrid approaches and ensemble learning have been used in recent studies to try to overcome these restrictions, however they often increase computer complexity and rely on manually created feature engineering. The suggested study, on the other hand, makes use of transformer-based architectures, such as ViT, Swin Transformer, DeiT, and T2T-ViT, which simulate global interactions over the entire image by using self-attention mechanisms. This capability shines when sorting cotton leaf issues, since texture shifts, warped veins, or odd colors often appear scattered across the surface. Earlier efforts usually tested one model setup—here, several transformers are measured side by side under identical conditions. What also sets this work apart lies in how it checks results. A lot of newer studies rely on just one training and testing division, which can give a shaky sense of reliability, especially if there aren’t many samples to begin with. To get around that issue—and keep class proportions steady while sharpening accuracy—the approach uses repeated stratified splits, cycling through five separate validation rounds. This validation strengthens credibility while broadening real-world relevance. Rather than pitting findings against unrelated data pools, the approach sidesteps shaky number-crunching, focusing instead on how methods shape outcomes. Context emerges through emphasis—on structural advantages, scrutiny in testing, and actual field utility—not just raw scores. Taken together, the transformer framework shows strong potential for spotting cotton leaf issues with consistency, supporting timely responses in farming systems. Multiple transformer variants were weighed with care, adding meaningful detail to ongoing exploration of visual models in smart crop monitoring.

## 5 Conclusion

This research introduced a transformer-driven system capable of automatically detecting cotton leaf illnesses through farm-related image data. Instead of traditional methods, it relied on Vision Transformers—paired with a thorough preparation process—to capture signs of disease in foliage images. For fair testing and even representation across categories, performance checks used stratified k-fold splits (ranging from k = 1 up to k = 5), alongside a separate reserve dataset. Results held strong throughout each fold, hitting a near-perfect 99.99% accuracy rate—not just once, but every time. This steady performance reveals the model’s toughness, proving it works where farming happens. Transformer-driven setups handle nearby and wide-ranging influences just right—key for telling plant illnesses apart accurately. All together, the study backs using high-level neural methods in smart farming, suggesting they might sharpen early warnings, guide choices, support healthier crops without waste. Coming work could pull in transparent AI tools, test smaller transformers on live data streams, adapt the setup to broader, richer collections of field records to make outputs clearer, earn grower confidence.

## Data Availability

The original contributions presented in the study are included in the article/supplementary material, further inquiries can be directed to the corresponding author.
